# The smoking and vaping model, A user-friendly model for examining the country-specific impact of nicotine VAPING product use: application to Germany

**DOI:** 10.1186/s12889-023-17152-y

**Published:** 2023-11-21

**Authors:** Luz María Sánchez-Romero, Alex C. Liber, Yameng Li, Zhe Yuan, Jamie Tam, Nargiz Travis, Jihyoun Jeon, Mona Issabakhsh, Rafael Meza, David T. Levy

**Affiliations:** 1 Lombardi Comprehensive Cancer Center, Georgetown University, 2115 Wisconsin Ave, suite 300, Washington, DC 20007 USA; 2 School of Public Health, Yale University, New Haven, CT USA; 3 Department of Epidemiology, University of Michigan, Ann Arbor, MI USA; 4 Integrative Oncology, BC Cancer Research Institute, British Columbia, Canada; 5 School of Population and Public Health, University of British Columbia, Columbia, Canada

**Keywords:** Tobacco smoking, Vaping, Prevalence, Computer simulation, Population health, Germany

## Abstract

**Background:**

Simulation models play an increasingly important role in tobacco control. Models examining the impact of nicotine vaping products (NVPs) and smoking tend to be highly specialized and inaccessible. We present the Smoking and Vaping Model (SAVM),a user-friendly cohort-based simulation model, adaptable to any country, that projects the public health impact of smokers switching to NVPs.

**Methods:**

SAVM compares two scenarios. The No-NVP scenario projects smoking rates in the absence of NVPs using population projections, deaths rates, life expectancy, and smoking prevalence. The NVP scenario models vaping prevalence and its impact on smoking once NVPs became popular. NVP use impact is estimated as the difference in smoking- and vaping-attributable deaths (SVADs) and life-years lost (LYLs) between the No-NVP and NVP scenarios. We illustrate SAVM’s adaptation to the German adult ages 18+ population, the Germany-SAVM by adjusting the model using population, mortality, smoking and NVP use data.

**Results:**

Assuming that the excess NVP mortality risk is 5% that of smoking, Germany-SAVM projected 4.7 million LYLs and almost 300,000 SVADs averted associated with NVP use from 2012 to 2060. Increasing the excess NVP mortality risk to 40% with other rates constant resulted in averted 2.8 million LYLs and 200,000 SVADs during the same period.

**Conclusions:**

SAVM enables non-modelers, policymakers, and other stakeholders to analyze the potential population health effects of NVP use and public health interventions.

**Supplementary Information:**

The online version contains supplementary material available at 10.1186/s12889-023-17152-y.

## Introduction

Nicotine Vaping Products (NVPs), or e-cigarettes, are changing the tobacco product landscape. Worldwide, 35 million individuals in 2019 were estimated to use NVPs and heated tobacco products, with these numbers increasing [1, 2]. NVPs are increasingly being used by smokers to quit [3–5], but there is concern about their increased use among youth [6].

NVPs deliver fewer toxicants than cigarettes [7–13] and efficiently deliver nicotine [14–18], thereby potentially serving as a “harm reducing” substitute for cigarettes. Exclusive NVP use reduces harm when used by those who would have instead initiated smoking or by former smokers who switched to NVPs and would not have otherwise quit smoking. NVP use increases harm if used by those who would not have otherwise started any combustible tobacco use or by former smokers who would have otherwise quit all combustible tobacco use [19].

Simulation models offer tools to examine potential public health implications of novel tobacco products like NVPs [20, 21]. They can also be used to identify evidence gaps needed to be filled to better understand the role of NVPs, and to develop effective tobacco control strategies [22, 23]. Globally, many countries are considering or have already restricted NVP availability [24] and could apply simulation models to help guide their decision-making [25]. While many models incorporate NVPs [26–34], most tend to be highly specialized, have substantial input data requirements, and are generally not accessible to researchers without modeling expertise.

The Smoking and Vaping Model (SAVM) adopts a public health framework [19] to assess the possible harm- increasing or -reducing of the use of NVPs in the population health. This framework is based on decision theory that considers the potential pathways of NVP and cigarette use by current, former and never cigarette users. It is based on previous work, which sets out a decision-theoretic analysis [19, 35]*.* SAVM was developed to be readily available and easily adaptable for non-modeling experts, and to facilitate outcome interpretation and transparency for all input parameters and methodologies. Based on a previous NVP replacement model [36], the SAVM offers greater flexibility by incorporating a wide range of cigarette and NVP initiation and cessation rates and switching parameters. It is developed in Microsoft Excel to remove technical barriers to implementation and facilitate adaptation for user-specific purposes.

Originally developed based on the US population [37], we now discuss the application of the SAVM to other countries. As an illustrative example, we present the model’s application for the German population: the Germany-SAVM. Germany was chosen as an example because there is evidence of increasing NVP consumption. This trend could potentially change because of Germany’s NVP permissive policies shifting toward stricter policies [38, 39]. Therefore, evidence on the impact of NVP use from SAVM could support NVP or smoking policy planning and surveillance in Germany. Prior tobacco simulation models for Germany have been used to evaluate smoking but not NVPs [40, 41]; the Germany-SAVM can facilitate decision-making in the era of NVP use.

## Methods

The SAVM is a cohort-based discrete-time simulation model that projects the impact of NVPs for specific birth cohorts in a given population. This approach recognizes that NVP use within a particular year depends on NVPs availability, NVP initiation and cessation rates, and the switching from smoking (i.e., cigarette use) to NVP use at specific ages. The SAVM simulates separate cohorts for males and females by individual age. The population evolves with age (i.e., yearly) and transitions between states are age and gender dependent. The model estimates the public health impact of NVP use by simulating and comparing two scenarios: the No-NVP and the NVP scenarios. The *No-NVP scenario* projects cigarette use and associated mortality outcomes in a country before NVP use became popular. Smoking rates evolve through smoking initiation and cessation. The NVP scenario estimates current and future smoking and vaping prevalence trends from the year that NVPs became more widely used. NVP use evolves through NVP initiation and cessation rates, and switching from cigarette to NVP use. The NVP-scenario for the US population has been previously validated agains US smoking and vaping prevalence [37]. The outcomes are derived based on the difference in smoking-attributable deaths (SADs) and smoking-and-vaping-attributable deaths (SVADs) and life years lost (LYLs) between the No-NVP and the NVP scenarios. SAVM’s pre-loaded input and transition parameters are based on US data. Adapting the model to other countries requires scaling US data to country-specific rates. However, the developer may substitute the pre-loaded US rates for country-specific rates if the information is available.

### Preliminary steps

First, SAVM requires the user to specify the *modeling period* (i.e., a maximum modeling period of 88 years) and *age ranges* (from 0 to 99 years) considered. The Germany-SAVM modeling period is set from 2012 to 2060.

The user then provides country-specific inputs for population projections, overall mortality, and life expectancy by age and gender for all modeling years (Table 1). German population projection data by single age, total mortality rates by five-year age groups for 2012 to 2060, and 2012 life expectancy were obtained from the United Nations World Population Prospects [42, 43].

**Table 1:** Data and parameters required for the smoking and vaping model

Input parameters	Description	Suggested data sources and pre-loaded estimate (Germany-SAVM)^b^
Population (*Compulsory user-input*)	Population by age, gender, and year	^b^**United Nations. World Population Prospects **[42]
Mortality rates (*Compulsory user-input*)	Overall mortality rates by age, gender, and year for never, current, and former smokers	World Health Organization. The Global Health Observatory [112] ^b^**United Nations. World Population Prospects 2019. Mortality Data **[43]
Life expectancy *(Compulsory user-input*)	Expected life years remaining of never smokers by age, gender, and year	Global Health Observatory. Life expectancy and healthy life expectancy [113] ^b^**United Nations. World Population Prospects 2019. Life expectancy at Birth **[43]
Smoking prevalence (*Compulsory user-input*)	Current and former smoking prevalence by age and gender for the initial year.	Global Health Observatory data repository [114] ^b^“**German Health Update**” (Gesundheit in Deutschlandaktuell, GEDA 2012–2013 [46]
Smoking initiation rate for the No-NVP scenario^a^	The proportion of male/female never smokers in year t at age a who initiate to current smokers in year t + 1 at age a + 1.	^b^CISNET Lung Group [61, 82, 115, 116], available on the CISNET website [117] *(Built-in data)*
Smoking cessation rate for the No-NVP scenario^a^	The proportion of male/female current smokers in year t at age a who quit smoking in year t + 1 at age a + 1. We consider net smoking cessation without any relapse.	^b^CISNET Lung Group [61, 82, 115, 116], available on the CISNET website [117]. *(Built-in data)*
NVP relative risk multiplier (*Optional-user input)*	Excess risk of NVP use measured relative to excess smoking risks (mortality rate of current smokers – mortality rate of never smokers)	NVP excess mortality risks set at 5%^b^ recommended ranges between 2% [80] and 50%.
NVP Switching rate	Rate of switching from smoking cigarettes to exclusive NVP use	Ranges from 0.6–4.0%, estimated by age group and gender using prospective analysis from PATH data 2013–2017 (*Built-in data*) ^b^ 50% of PATH’s switching rates
Rate of decline in the NVP switching rate	Yearly exponential process (annual change rate) to allow for constant relative change in the individual	Set at 90%, implying a 10% annual reduction in the NVP switching rate over time Suggested annual rate − 50 to 103%^c^
Smoking initiation multiplier in the NVP Scenario (*Optional-user input)*	Ratio of smoking initiation rate in the NVP Scenario to smoking initiation rate in the No-NVP Scenario	75% of the No-NVP smoking initiation rate, based on recent studies [67–69]. *(Built-in data)* ^b^ 88% of the No-NVP smoking initiation rate Suggested annual change rate 50 to + 150%^c^
NVP initiation multiplier in the NVP Scenario (*Optional-user input)*	Ratio of NVP initiation rate in the NVP Scenario to smoking initiation rate in the No-NVP Scenario	50% of the No-NVP smoking initiation rate, based on recent studies [118–121]. *(Built-in data)* ^b^ 25% of the No-NVP smoking initiation rate Suggested annual change rate: 50 to + 150%^c^
Smoking cessation multiplier in the NVP Scenario (*Optional-user input)*	Ratio of smoking cessation rate in the NVP Scenario to smoking cessation rate in the No-NVP Scenario	^b^100% of the No-NVP smoking cessation rate *(Built-in data* Suggested annual change rate 50 to + 150%^c^
NVP cessation multiplier in the NVP Scenario (*Optional-user input)*	Ratio of NVP cessation rate in the NVP Scenario to smoking cessation rate in the No-NVP Scenario	^b^100% of the No-NVP smoking cessation rate *(Built-in data* Suggested annual change rate: 50 to + 150%^c^

^a^*Smoking initiation and cessation rates* are pre-loaded parameters using US data and do not need to be provided by the user unless country-specific parameters are desired. *NVP =* nicotine vaping product, *No-NVP Scenario* = values in the absence of NVP use. *NVP Scenario* = values with NVP use. PATH = The Population Assessment of Tobacco and Health Study, US.^b^ data source used for the Germany-SAVM. ^c^*Suggested annual change rates* higher or lower can result in initiation rates above 100% and cessation rates above 50%. **Bold** Germany specific data sources

Since Germany’s overall mortality data was only available by five-year age groups, SAVM transformed that data into single ages by assuming the same death rates for each age within a given age group (SAVM User Guide, sec 3.2.2) [44]. The 2012 overall life expectancy was transformed into never smokers’ life expectancy for each modeling year by multiplying the age and gender-specific life expectancy of US never smokers by the ratio of the 2012 German/US overall life expectancy [45], assuming that each individual age-gender ratio is constant over the modeling period (see SAVM User Guide, sec 3.2.3) [44].

### No-NVP scenario

**Fig. 1:**
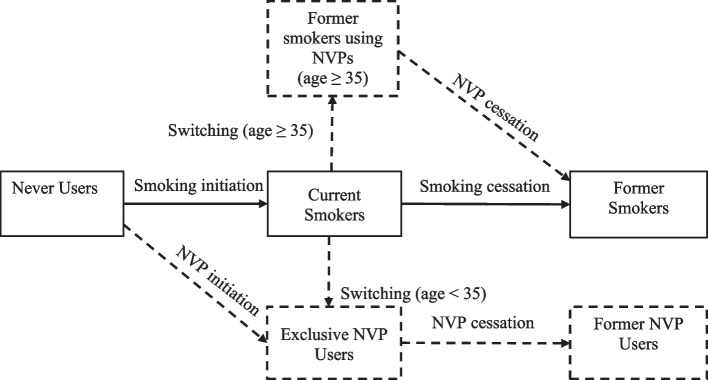
Smoking and NVP Use Transitions in the No-NVP and NVP Scenarios

The No-NVP scenario projects the prevalence and mortality of never, current, and former smokers over time in the absence of NVPs (Fig. 1). The user enters country-specific never, current, and former smoking prevalence for the first year of the modeling period by age and gender. The smoking prevalence should reflect regular or established cigarette use (i.e., smoked at least 100 cigarettes during their lifetime and currently smokes every day or somedays) since regular use is more directly relevant to public health outcomes than occasional use (see Supplementary Table 1 for recommended definitions) [19].

The Germany-SAVM uses smoking prevalence from the “German Health Update” (Gesundheit in Deutschland aktuell [GEDA]) 2012 [46]. Five different German surveys with smoking data were considered (GEDA, the German Health Interview and Examination Survey for Adults [DEGS], Microcensus, The German Study on Tobacco Use [DEBRA], and Eurobarometer) for model building and validation. GEDA was chosen for model building because it is one of the largest nationally representative surveys, and data was available for 2012 before significant NVP use in Germany [47–49].

GEDA defines current smokers as those who smoke any combustibles, including manufactured cigarettes, hand-rolled cigarettes, cigars, or pipes (daily or occasionally). The data were obtained by age group (18–29, 30–44, 45–64, and 65–99 years old) combined for East and West Germany. Current smoking prevalence which includes any combustible tobacco use, was 27.5% in 2012 in Germany [46], which was higher than every day or someday use of any combustible tobacco (22.2%) for that same year in the US [50]: (Germany vs. US: 34.7% vs. 21.3% for ages 18–29; 34.5% vs. 27.0% for ages 30–44; 30.4% vs. 22.1% for ages 45–64, and 12.0% vs. 9.4% for ages 65+). We transformed age-group prevalence into individual age prevalence using the midpoints and then smoothing the estimates to represent the data distribution. (SAVM User Guide, sec 3.2.4). For Germany-SAVM, we apply the same prevalence for ages 18 to 22 as the prevalence at age 23 (the first midpoint in the 18–29 age group from GEDA) and the same prevalence for the upper age ranges (83–99 years) as the prevalence at age 82 (the last midpoint in the 65–99 age group from GEDA).

Smoking prevalence evolves based on initiation and cessation rates. These rates are set as a linear function of US rates and scaled for the initial modeling year’s country-specific/US smoking prevalence ratio. For Germany-SAVM, we applied the ratio of German to US smoking prevalence for ages 18–24 in 2012 to US initiation rates at all ages and the ratio of German to US smoking prevalence for ages 25–64 in 2012 to US cessation rates at all ages. Most relapse by former smokers occurs within 1 year after quitting. Relapse among those who quit for ≥2 years is less than 30%” [51–54]. The model’s cessation rate collapse cessation and relapse into a measure of cessation net of relapse. SAVM cessation rate is based on former smokers who quit for at least 2 years and is meant to reflect permanent cessation [55]. (SAVM User Guide, sec 3.2.5).

To derive country-specific mortality rates by smoking status (never, current and former), SAVM multiplies US death rates by smoking status to the ratio of the country/US overall mortality rates. For smokers who quit before age 35, SAVM assumes the same mortality risks as never smokers [56–58]. SAVM’s US smoking status and mortality data (i.e., prevalence, mortality, and life expectancy) are obtained from the US National Cancer Institute, Cancer Intervention and Surveillance Modeling Network (CISNET) Lung group [56, 59–61]. The CISNET smoking status estimates are based on age-period-cohort models using data from the US National Health Interview Survey from 1965 to 2018: this captures trends in smoking before NVP use became more prevalent in the US [62]. Similarly, the CISNET mortality rates are based on mortality data through 2012 and smoking relative risks informed by data through the early 2000s, priot to the wide adoption of NVPs.

### NVP scenario

The NVP scenario projects the prevalence of current, former, and never smokers plus three new categories: current exclusive NVP users, former smokers using NVPs, and former NVP users (Fig. 1). Exclusive NVP users are those who initiate NVP use in the absence of smoking and those who switch to vaping from smoking before age 35. Former smokers using NVPs are those who quit cigarette use after age 34 and switch to exclusive NVP use. Dual users (of both cigarettes and NVPs) are considered current smokers and assumed to have the same mortality risks as exclusive cigarette users [12, 13] (Supplementary Table 1).

The NVP scenario considers five types of transition parameters: (1) switching from smoking to vaping, (2) vaping initiation, (3) smoking initiation, (4) vaping cessation, and (5) smoking cessation (Table 1). The model defines the last four transition parameters at levels proportional (i.e., as multipliers) to the respective transition parameters in the No-NVP scenario. The model allows NVP scenario transition parameters to vary over time by enabling a yearly exponential process (annual change rate) in the individual parameters (SAVM User Guide, sec 3.3.4).

For the Germany-SAVM, pre-loaded transitions rates and multipliers estimates were modified to account for the difference in NVP use rates between Germany and US. While any combustible use is higher in Germany than in the US, as indicated above, the opposite is observed for German NVP (e-cigarette) prevalence. In 2012, prevalence of NVP ever-use in the German population aged 15 + was 20.2% (95% CI 15.6–24.9%) [63]. In 2016–17, current NVP use prevalence in the German 14+ population was 1.9% (males 2.6% and females 1.3%) with the highest use in individuals ages 18–24 (3.5%) [64]. In contrast, in the US, by 2014, ever-NVP use in the 18+ population was 12.6%, and current NVP use was 3.7% [65]. In 2017, US current NVP use in adults 18+ was 2.8%, (males 3.2% and females 2.4%) with the highest use in the 18–24 age group (5.2%) [66].

Direct transitions from cigarette to exclusive NVP use occur through a constant NVP switching parameter, starting at the base year of the modeling period (e.g., Germany-SAVM at 2012 from the 2012–2060 period). Pre-loaded SAVM switching rates were derived from the US Population Assessment of Tobacco and Health (PATH) Study Waves 1–4 (2013–2017) (Supplementary Table 2). To account for differences between German and US smoking and NVP prevalence, we reduced SAVM’s pre-loaded switching rates by 50% and allowed a 10% annual reduction rate starting in 2018.

The smoking and NVP initiation rates in the NVP scenario are developed by applying a separate cigarette and NVP use multiplier to the No-NVP smoking initiation rates. SAVM’s US initiation multipliers are set as 75% for smoking and 50% for NVP use of the No-NVP smoking initiation rate for both genders and all ages and are assumed constant over time. The values are based on the rapid decline in US youth and young adult smoking since vaping increased [67–69]. However, the German population’s smoking initiation starts at younger ages compared to the US. In 2012 in Germany, most of the initiation into regular smoking occurred between ages 15 to 17 [70, 71]. For that same year, the US reported the highest average age of daily cigarette use initiation between 18 and 21 years, followed by 15 to 17 years [72]. To account for these differences, the Germany-SAVM smoking and NVP initiation multipliers were set as 88 and 25%, respectively (Table 1).

Similar to initiation rates, the smoking and NVP cessation rates apply a pattern relative to smoking cessation in the No-NVP scenario. NVPs may influence smoking outcomes through cessation from both smoking and vaping, and through smoking initiation and switching. Considerable evidence indicates that NVPs may improve cessation from smoking and could be easier to quit than smoking [73–76]. Still, we set smoking and NVP cessation rates in the NVP scenario in the US-SAVM and the Germany-SAVM models to the same rate as the No-NVP smoking cessation rates (equal to 100% of the No-NVP smoking cessation rate) for both genders and all ages; they are assumed constant over time in this analysis but may be adjusted by the user to incorporate trends. We note that smokers may also quit smoking but continue to vape via the switching parameter. We have applied this method for simulation exercises for other countries, including Australia and Canada [77, 78].

An NVP *relative risk* multiplier specifies the NVP mortality risks *relative to* the excess mortality risk for current and former smokers (*current or former smoker death rate – never smoker death rate*). By default, SAVM specifies a constant current and former NVP users mortality risks at 5% of that of smoking for both genders at all ages, based on estimates from a multi-criteria decision analysis [79] and an independent review [80]. Germany-SAVM applies an initial mortality relative risk of 5%. As this estimate may be debatable [81], we conduct sensitivity analyses and recommend that the user consider lower and upper-end mortality estimates between 2.5 and 50%.

### Model outputs

SAVM estimates public health impacts in terms of smoking-attributable deaths (SADs), smoking and vaping attributable deaths (SVADs), and life-years-lost (LYLs). Based on previous approaches [60, 82], SADs and SVADs are calculated by multiplying the excess mortality of current and former smokers or NVP users by the number of individuals in their smoking or NVP use category, and LYLs are calculated at each age by multiplying the number of SADs or SVADs by the life-years remaining of a never smoker. The public health impact of NVP use is evaluated as the difference in SVADs and LYLs between the No-NVP and NVP scenarios.

### Germany-SAVM validation and sensitivity analyses

Because prevalence rates for the same year often vary from survey to survey and are only available for limited years, we validate SAVM by comparing relative changes in smoking prevalence. The validation data used for the Germany-SAVM was over a specified period based on survey data availability. The Germany-SAVM was first validated by comparing relative changes in current smoking prevalence from the NVP scenario to those from Eurobarometer [83] from 2014 to 2020. Eurobarometer is a survey conducted yearly since 2014 by the European Union (EU) Institutions to monitor Europeans’ attitudes towards various topics of interest. Current smokers in Eurobarometer were defined as “currently smoke” cigarettes, cigars, cigarillos, or a pipe. This current smoker definition matched well with the definition used in the GEDA survey. The model’s smoking prevalence was also validated against the German Microcensus 2017 [84], a nationally representative survey that collects tobacco use data using similar methods and smoking definitions as the GEDA.

Finally, we validated NVP use by comparing the 2017 SAVM NVP use estimates against results from The German Study on Tobacco Use (Deutsche Befragung zum Rauchverhalten, [DEBRA]) 2016–2021 for both genders combined [64, 85]. DEBRA survey collects information on NVP use from nearly 2000 individuals ages 14+ every 2 months. This survey was chosen because data were available in successive years after NVPs became more widely used, and it allows comparisons by age group and gender.

We conducted sensitivity analyses with the NVP relative risks at 5 and 40% of the excess smoking risk and over plausible ranges of +/− 50% of the NVP switching rates and initiation and cessation multiplier parameters. Recommended rates for sensitivity analyses are provided in Table 1.

## Germany-SAVM results

### Validation of smoking prevalence

Compared to Eurobarometer data, for ages 15+, Germany-SAVM projected that male (female) smoking prevalence declined from 29.1% (22.5%) in 2014 to 24.7% (19.6%) in 2020, while Eurobarometer reported a decline from 33.83% (20.8%) in 2014 to 27.6% (18.8%) in 2020. Over 2014–2020, Germany-SAVM projects an overall smaller relative reduction in smoking prevalence of 15.1% (12.9%) compared to 18.5% (9.9%) from the Eurobarometer. Eurobarometer data showed a more rapid decline in Germany’s male smoking prevalence between 2014 and 2018 than SAVM, the period when NVP use was increasing. However, Germany’s male smoking prevalence increased from 2018 to 2020, moving the relative declines in smoking prevalence much closer to SAVM projections. In contrast, Eurobarometer showed an increase in female smoking prevalence from 2014 to 2018, followed by a decrease from 2018 to 2020 (Supplementary Table 3).

We also validated SAVM smoking prevalence by age group against the German Microcensus for 2017. Germany-SAVM slightly overestimated the smoking prevalence for adults ages 18+ by a relative difference of 3.8% (SAVM 27.4% vs. Microcensus 26.4%) for males and a relative difference of 16% (SAVM 21.5% vs. Microcensus 18.6%) for females. For males ages 18 to 64 years, SAVM underpredicted smoking prevalence with relative differences between estimates ranging from 24.4% to − 0.1% but overestimated for males aged 65 to 70 with differences ranging from 18.4 to 37.1%. (Supplementary Fig. 1a) For females, in general, SAVM overestimated smoking prevalence with relative differences between SAVM and the Microcensus from 3.7 to 23.0% for ages 20 to 65 years. SAVM underestimated prevalence for individuals ages 18 to 20 years and 50 to 60 years. (Supplementary Fig. 1b). The largest relative differences between SAVM and the Microcensus were observed for ages 75+ for males and 70+ for females.

### Validation of NVP prevalence

For ages 18–24, the DEBRA survey and Germany-SAVM both reported current NVP prevalence at about 4% in 2016; DEBRA remained at 4% through 2018, while SAVM increased to 5.5% in 2019. After 2019, DEBRA NVP use fell to 2%, while SAVM increased to 6.5% in 2021. In 2016, for ages 25+, the NVP prevalence was 1% in SAVM compared to 1.5% in DEBRA, and both were at 1.5% in 2019. However, in 2021, SAVM increased to 2.5%, while DEBRA fell to 1%. (Supplementary Figs. 2a and b).

### Public health impact under the baseline NVP compared to the no-NVP scenario

Table 2 presents the Germany-SAVM results for all cohorts for the modeling period 2012–2060. In the No-NVP scenario, smoking prevalence among adult ages 18+ is projected to decline from 32.1 to 20.6% for males and from 24.7 to 16.1% for females from 2012 to 2060, which is about 35% relative reduction in smoking combustible tobacco products for both genders. In the No-NVP scenario, from 2012 to 2060, SAVM projects 5.0 million SADs with 46.4 million LYLs in males and 2.2 million SADs with 18.5 million LYLs in females.

**Table 2:** The Germany SAVM model estimates for all cohorts (ages 18–99) with new births for 2012–2060. NVP risks at 5% those of excess smoking risks

Year		2012	2017	2040	2060	Cumulative^a^
**Male**						
**No-NVP scenario**^**b**^	Smokers	32.1%	29.1%	22.5%	20.6%	–
	SADs	111,909	108,729	99,458	86,383	4,956,100
	LYLs	1,171,679	1,189,823	890,229	671,585	46,425,200
**NVP Scenario**^**c**^	Smokers	32.1%	27.4%	18.1%	16.6%	–
	NVP users	0.0%	1.3%	4.4%	5.5%	–
	FS-NVP users	0.0%	0.8%	1.3%	0.5%	–
	SVADs	111,909	107,342	94,707	79,977	4,757,770
	LYLs	1,171,679	1,164,455	805,087	567,618	42,904,963
**Net impact**^**d**^	Deaths averted	0	1386	4751	6406	198,330
	LYLs averted	0	25,367	85,142	103,967	3,520,237
**Female**						
**No-NVP scenario**^**b**^	Smokers	24.7%	22.6%	17.7%	16.1%	–
	SADs	52,058	45,517	44,666	40,169	2,195,568
	LYLs	440,936	441,049	369,645	267,812	18,462,657
**NVP Scenario**^**c**^	Smokers	24.7%	21.5%	15.0%	13.4%	–
	NVP users	0.0%	0.7%	2.8%	3.9%	–
	FS-NVP users	0.0%	0.55%	0.8%	0.3%	–
	SVADs	52,058	44,611	42,352	37,872	2,101,907
	LYLs	440,936	429,620	340,489	237,606	17,247,469
**Net impact**^**d**^	Deaths averted	0	906	2314	2296	93,661
	LYLs averted	0	11,428	29,156	30,206	1,215,187
**Both genders**						
**Net impact**^**d**^	Deaths averted	0	2292	7065	8702	291,991
	LYLs averted	0	36,795	114,298	134,173	4,735,424
	Deaths averted	0.0%	1.5%	4.9%	6.9%	4.1%
	LYLs averted	0.0%	2.3%	9.1%	14.3%	7.3%

Results for the NVP scenario are estimated applying a 5% NVP mortality risk of that from smoking and with smoking initiation at 88%, NVP initiation at 25%, and cessation for smoking and NVP at 100% from that of the No-NVP scenario

*NVP* nicotine vaping product, *FS-NVP* former smokers nicotine vaping users, *SADs* smoking-attributable deaths, *SVADs* smoking and vaping attributable deaths, *LYL* Life years lost

^a^Cumulative results include the deaths and life-years lost, which are the sum of attributable deaths or life-years lost over the years 2012–2060

^b^No-NVP Scenario refers to values in the absence of NVP use

^c^NVP scenario refers to values with NVP use

^d^ Net impact is the difference between the No-NVP Scenario and NVP Scenario in deaths averted (SADs-SVADs) and LYLs

§ The relative net impact (%) in averted deaths and LYLs are calculated for both genders as:

Deaths averted (%) = Deaths averted /SADs _No-NVP_; LYLs averted (%) = LYLs averted /LYLs _No-_

In the NVP scenario, SAVM projected that smoking prevalence for ages 18+ declines to 16.6% for males and 13.4% for females by 2060, which is about 48 and 46% relative reduction in smoking prevalence for males and females, respectively. Exclusive NVP prevalence increases between 2017 and 2060 from 1.3 to 5.5% for males and from 0.7 to 3.9% for females. From 2012 to 2060, SAVM projects 4.8 million SVADs with 42.9 million LYLs in males and 2.1 million SVADs with 17.3 million LYLs in females in the NVP scenario.

Compared to the No-NVP scenario, the estimated public health net impact of NVPs by 2060 is a reduction in relative terms of the smoking prevalence of 19% [(16.6–20.6)/20.6] for males and 17% [(13.4–16.1)/16.1] for females. In addition, the use of NVPs is projected to avert 0.3 million premature deaths and secure 4.7 million life-years by 2060, representing relative reductions of 4.1% in cumulative premature deaths and 7.3% in LYLs in German population.

### Sensitivity analysis of NVP transition parameters

**Fig. 2:**
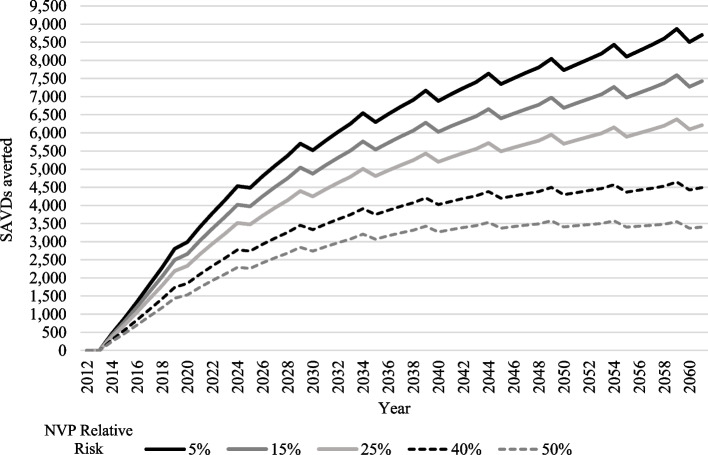
Estimated adverted smoking and vaping-attributable deaths applying different values for the NVP excess mortality risk. Results from the sensitivity analysis for both genders ages 8–99 years from 2012 to 2060

Compared to the 0.3 million SVADs and 4.7 million LYLs between 2012 and 2060 under the base case NVP scenario, averted SVADs and LYLs fell to 0.17 million and 2.8 million, respectively, with a 40% relative excess mortality risk while holding transition parameters constant. Less conservative scenarios such as a 15% relative risk projected 0.25 million fewer deaths and a 25% relative risk projected 0.22 million fewer deaths. However, with more pessimistic scenarios such as 50% relative risk (0.13 million SAVDs averted), the model shows that a point of inflection is reached after 2040 where the increase in deaths averted is minimal. (Fig. 2).

When applying variations in transition parameters, SAVM projected the highest number of averted SVADs and LYLs with the smoking cessation multiplier at 150% of its original value, 0.97 million SVADs and 12.0 million LYLs with a 5% excess NVP risk. These estimates decreased to 0.89 million SVADS and 10.1 million LYLs with a 40% excess NVP risk. Large reductions were observed when increasing the switching rate by 150% with a 10% annual decay and reducing smoking initiation by 50%. In contrast, Germany-SAVM estimated an increase in SVADs and LYLs when increasing the cigarette initiation multiplier by 150% and reducing the switching rate and cessation multiplier by 50% (Tables 3 and 4).

**Table 3:** Estimated adverted smoking-attributable deaths applying different values for the NVP Scenario input parameters. Results from the sensitivity analysis for both genders ages 8–99 years from 2012 to 2060

Scenario	NVP relative risk^**a**^ (Risk _**NVP)=**_5%		NVP relative risk^**a**^ (Risk _**NVP)=**_40%		Relative Change 5% vs. 40%^**d**^
**No-NVP Scenario**	**Total SVADs**		**Total SVADs**		**–**
	7,151,668		7,151,668		–
**NVP Scenario with parameter changes from base model**	**Averted SVADs**^**b**^	**Relative Difference (vs. base model estimate)**^**c**^	**Averted SVADs**^**b**^	**Relative Difference(vs. base model estimate)** ^**c**^	**–**
Base model estimate^e^	291,991	0.0%	167,127	0.0%	−42.8%
Switching rate and annual change					
50% of switching^f^, 10% annual decay^g^	160,588	−45.0%	89,496	−46.5%	−44.3%
150% of switching, 10% annual decay	414,852	42.1%	239,723	43.4%	−42.2%
Initiation rate multipliers and annual change					
50% smoking initiation^h^, no annual decay	345,646	18.4%	222,726	33.3%	−35.6%
150% smoking initiation, no annual decay	245,410	−16.0%	118,780	−28.9%	−51.6%
50% NVP initiation^i^, no annual decay	290,608	−0.5%	172,263	3.1%	−40.7%
150% NVP initiation, no annual decay	293,338	0.5%	162,200	−2.9%	−44.7%
Cessation rate multipliers and annual change					
50% of smoking cessation multiplier^j^	− 769,218	− 363.4%	− 906,453	− 642.4%	17.8%
150% of smoking cessation multiplier	972,300	233.0%	857,132	412.9%	−11.8%
50% of NVP cessation^k^ multiplier	284,899	−2.4%	116,621	−30.2%	−59.1%
150% of NVP cessation multiplier	296,656	1.6%	200,873	20.2%	−32.3%

NVP nicotine vaping product, Smoking and Vaping attributable deaths (SVADs)

^a^ The NVP relative risk multiplier is the mortality risk of NVPs as a percentage of the excess mortality risk of smoking

^b^ The absolute reduction in SVADs in the NVP Scenario compared with the No-NVP Scenario over 2012–2060

^c^ The relative percent change in averted SVADS for each NVP Scenario is compared with the initial NVP Scenario (best estimate). A negative (positive) value implies that changing the parameter will decrease (increase) the averted SAVDs in the specific scenario relative to averted SVADs in the initial NVP Scenario

^d^ The relative percent change in averted LYLs between scenarios with NVP risk multipliers of 5% vs. 40% is calculated as (Averted SVADs with 40% NVP risk −Averted SVADs with 5% NVP risk)/Averted SVADs with 5% NVP risk

^e^ The initial values for each input parameter in the NVP Scenario are as follows. NVP switching rate for males (females): 4% (2.5%) for ages 24 and below, 2.5% (2.0%) for ages 25–34, 2.5% (1.6%) for age 35–44, 1.3% (1.4%) for ages 45–54, 1.2% (1.4%) for ages 55–64, and 0.6% (1.0%) for ages 65 and above; smoking initiation multiplier = 88%; NVP initiation multiplier = 25%; Smoking cessation multiplier = NVP cessation multiplier =100%

^f^ NVP switching rate is the annual rate at which current smokers switch to NVPs

^g^ Annual decay rate is the exponential rate of decline in switching rates over time

^h^ Smoking initiation multiplier is relative to smoking initiation in the No-NVP Scenario

^i^ NVP initiation multiplier is relative to smoking initiation rates in the No-NVP Scenario

^j^ Smoking cessation multiplier is relative to smoking cessation in the No-NVP Scenario

^k^ NVP cessation multiplier is relative to smoking cessation rates in the No-NVP Scenario

**Table 4:** Estimated life-years saved applying different values for the NVP Scenario input parameters. Results from the sensitivity analysis for both genders 18–99 from 2012 to 2060

Scenario	NVP relative risk^**a**^ (Risk _**NVP)=**_5%		NVP relative risk^**a**^ (Risk _**NVP)=**_40%		Relative Change 5% vs. 40%
**No-NVP Scenario**	**Total LYLs**		**Total LYLs**		**–**
	64,887,857		64,887,857		–
**NVP Scenario with parameter changes from base model**	**Averted LYLs**^**b**^	**Relative Difference**^**c**^	**Averted LYLs**^**b**^	**Relative Difference**^**c**^	**–**
Base model estimate^e^	4,735,424	0.0%	2,745,088	0.0%	−42.8%
Switching rate and annual change					
50% of switching^f^, 10% annual decay^g^	2,737,897	−42.2%	1,517,938	−44.7%	−44.6%
150% of switching, 10% annual decay	6,589,927	39.2%	3,884,888	41.5%	−41.0%
Initiation rate multipliers and annual change					
50% smoking initiation^h^, no annual decay	6,282,285	32.7%	4,343,035	58.2%	−30.9%
150% smoking initiation, no annual decay	3,412,112	−27.9%	1,375,850	−49.9%	−59.7%
50% NVP initiation^i^, no annual decay	4,688,798	−1.0%	2,885,784	5.1%	−38.5%
150% NVP initiation, no annual decay	4,780,843	1.0%	2,610,916	−4.9%	−45.4%
Cessation rate multipliers and annual change					
50% of smoking cessation multiplier^j^	−5,390,126	− 213.8%	−7,516,335	− 373.8%	39.4%
150% of smoking cessation multiplier	11,957,832	152.5%	10,081,676	267.3%	−15.7%
50% of NVP cessation^k^ multiplier	4,658,618	−1.6%	2,156,853	−21.4%	−53.7%
150% of NVP cessation multiplier	4,791,564	1.2%	3,177,221	15.7%	−33.7%

NVP nicotine vaping product, LYLs life-years lost

^a^ The NVP relative risk multiplier is the mortality risk of NVPs as a percentage of the excess mortality risk of smoking

^b^ The absolute reduction in life-years lost in the NVP Scenario compared with the No-NVP Scenario over 2012–2060

^c^ The relative percent change in averted LYLs for each NVP Scenario is compared with the initial NVP Scenario (best estimate). A negative (positive) value implies that changing the parameter will decrease (increase) the averted LYLs in the specific scenario relative to averted LYLs in the initial NVP Scenario

^d^ The relative percent change in averted LYLs between scenarios with NVP risk multipliers of 5% vs. 40% is calculated as (Averted LYLs with 40% NVP risk −Averted LYLs with 5% NVP risk)/Averted LYLs with 5% NVP risk

^e^ The initial values for each input parameter in the NVP Scenario are as follows. NVP switching rate for males (females): 4% (2.5%) for ages 24 and below, 2.5% (2.0%) for ages 25–34, 2.5% (1.6%) for age 35–44, 1.3% (1.4%) for ages 45–54, 1.2% (1.4%) for ages 55–64, and 0.6% (1.0%) for ages 65 and above; smoking initiation multiplier = 88%; NVP initiation multiplier = 25%; Smoking cessation multiplier = NVP cessation multiplier =100%

^f^ NVP switching rate is the annual rate at which current smokers switch to NVPs

^g^ Annual decay rate is the exponential rate of decline in switching rates over time

^h^ Smoking initiation multiplier is relative to smoking initiation in the No-NVP Scenario

^i^ NVP initiation multiplier is relative to smoking initiation rates in the No-NVP Scenario

^j^ Smoking cessation multiplier is relative to smoking cessation in the No-NVP Scenario

^k^ NVP cessation multiplier is relative to smoking cessation rates in the No-NVP Scenario

## Discussion

The SAVM provides users with a tool to examine the potential impact of NVP use on smoking and vaping behaviors and subsequent mortality outcomes. We developed the SAVM to provide a model that can be applied by those less experienced in modeling. The population, health outcomes, and smoking prevalence data needed for the model are minimal and these data can generally be found through publicly available sources. We built the Germany-SAVM using publicly available country specific data from the WHO and United Nations databases and smoking prevalence from the GEDA, a nationally representative survey. Using these three data sources, SAVM’s formulas allow us to transform US pre-loaded model parameters into Germany-specific inputs and estimate the potential public health impact of NVP use for the German population. Although the SAVM is accessible to people who do not have much experience in simulation modeling, it also allows advanced modelers to modify underlying input data, equations, and its structures [44]. Nonetheless, the simplicity and transparency of SAVM has some drawbacks such as its inability to account for interactions between other risk factors or products that could influence the outcome and its limited number of possible outcomes.

Germany-SAVM estimated a reduction of 4.7 million life-years lost and almost 300,000 deaths averted associated with NVP use. However, the estimates should be viewed cautiously since model validation indicated that smoking and NVP prevalence were overestimated, especially for ages 18–24 female, and thus may underestimate the impacts of NVP since smoking prevalence has been overestimated.

The lack of better validation may reflect uncertainty about German tobacco use behaviors, particularly regarding switching from cigarette to NVPs or other products, since survey data to validate the model was limited. Ideally, SAVM estimates should be compared against various years of nationally representative smoking and NVP prevalence by age group and gender from the same survey, preferably the one used as input. After 2012, GEDA only collected information for 2019/2020, the database was not publicly available at the time of this study, and publications did not report the necessary data. We were also unable to find other German nationally representative population surveys with the required characteristics. Consequently, we validated the Germany-SAVM against three surveys: the Microcensus, Eurobarometer, and DEBRA. The Microcensus and Eurobarometer allowed us to compare smoking population estimates by age group and gender. The Microcensus is a nationally representative survey, and its large sample size enables more accurate age group comparisons. Eurobarometer provided consistent yearly post-NVP era smoking data. Because these surveys did not provide sufficient NVP use data for validation, we decided to use the DEBRA survey to validate Germany-SAVM NVP estimates. DEBRA is a nationally-representative household survey that permits comparison across different years of NVP use data and limited age groups. Continuous data, preferably longitudinal data is needed to better understand the relevant transitions. Application of the model may help policy makers recognize the limitations of current surveys for effective monitoring of product use behaviors.

## Limitations

SAVM’s accuracy depends on the availability of tobacco surveillance data and how the data exhibit changes in smoking behaviors associated with the country’s tobacco regulations. Germany’s limited smoking and NVP data sources, and their low frequency of data collection on tobacco control, restricted our ability to adapt Germany-SAVM to represent product use trends that reflect the current German regulatory scenario. SAVM rescaled US data to adapt to other countries, differences between collecting data methods between countries may influence model validation. German surveys smoking prevalence definition includes every day or occasional use of any combustible tobacco, and US-SAVM current smoking definition refers to individuals who have smoked at least 100 cigarettes in their lifetime. However, 2010 data reported that 83% of females and 76% of males of all combustible tobacco users (smokers) in Germany are cigarette users [86]. Still, even same product use definition can produce different population estimates for the same year of data collection [87]. Our analysis did not consider heated tobacco products, although their role appears minor [88, 89]. The failure to accurately estimate the decline in NVP use and the leveling off from combustible tobacco use since 2018 may also reflect a failure to incorporate changes in tobacco regulations into the model. Since 2018, considerable publicity has been devoted to the potential harms of NVPs, and German states and the federal government have implemented stricter NVP regulations. This has been augmented by the passage of the 2021 Tobacco Duty Modernization Act (Tabaksteuermodernisierungsgesetz )[38], which increased the excise tax rates for cigarettes, NVPs, and heated tobacco products.

The SAVM results should also be evaluated in the context of the model’s assumptions and data inputs (see Supplementary Table 5). For instance, SAVM assumes that former smokers’ mortality risk is dependent on age and birth cohort year [90, 91], but it has been observed that mortality risk from smoking to be inversely associated with quit years falling close to the same level of risk as never smokers after 10 years of quit [92–96]. Further, NVP is a novel product and there is a lack of evidence on long-term impact of NVP use, dual usage or quit years on mortality or associated chronic disease development. We assume that the risk for former NVP users is reduced by the ratio of former to current smoker risk, using the same risk of 5% for former and current NVP users. NVP mortality risk can be updated as evidence evolves. The No-NVP scenario refers to data through 2012 and assumes trends in smoking up until that year would have continued in the absence of NVPs. However, the counterfactual is unknown; post-2012 smoking prevalence declines may have accelerated or decelerated without NVPs at a different rate in Germany than in the US. The model relies on underlying US smoking trends to project future smoking and mortality rates for other countries. For the Germany-SAVM, we modified the transition parameters to adapt to German tobacco use patterns. Still, the shortage of information on transitions between smoking and NVP use for the German population and the effect of tobacco control policies on behaviors may have impaired Germany-SAVM from reflecting pre-NVP trends.

Parameters in the NVP scenario may also affect the validity of the model. Compared to cigarettes, NVPs are newer products with less empirical evidence about their health effects and usage/transition patterns [97, 98] The true extent of NVP health risks is not well understood [81]. We performed sensitivity analyses increasing the NVP excess risk parameter from 5,15, 25, 40 and 50%, to consider a range of potential effects. We observed that a conservative approach of 15% % NVP risk (Supplementary Table 4), provides estimates similar to the 5% risk case. When applying 40% or higher, the number of deaths averted becomes stagnant (Fig. 2).

In addition, NVPs’ disruption of the tobacco landscape depends on each country’s existing social and regulatory environment, for NVPs and cigarettes and other tobacco products [99, 100]. Consequently, patterns of future NVP use are challenging to predict because they depend on policies that encourage the growth (or decline) of the NVP market. SAVM users are encouraged to conduct sensitivity analyses on NVP risks and the NVP scenario transition rates and multipliers to investigate the influence of NVP use under a range of plausible parameters. We suggest such ranges in the SAVM User Guide [44].

The SAVM treats those who experimented with or use NVPs only occasionally as never smokers. It also treats current smokers who use NVPs temporarily (as a dual user or exclusive user) and quit both cigarettes and NVPs products within 2 years as former smokers. The potential gateway effect of vaping to smoking occurs through the net NVP and smoking initiation. Because transitions to dual use are often temporary [101–104] and dual users have risks comparable to smokers [12, 13], we chose not to distinguish between exclusive smokers and dual users in the NVP scenario. This assumption implies that dual users will have the same cessation and switching patterns with the same health risks as exclusive smokers. However, the sensitivity analysis allows to consider modifications in smoking behavior parameters that could implicit allow the effect of dual use. Should research demonstrate dual users to have a different health profile from exclusive smokers, the SAVM can be easily modified accordingly.

The SAVM is designed to simulate patterns of switching and substitution between cigarettes and NVPs. The model structure and design are flexible enough to simulate their specific use patterns if the user aims to model a specific NVP or any new emerging tobacco prodcuts, such as heated tobacco product (HTP) [86–88]. When including other tobacco products use into the current SAVM, data-driven parameters for NVPs may not be generalizable to other products. One way to handle such case is that for example, the user may include NVPs and HTPs in the same category but considerable attention must be made to the complex substitution patterns between cigarettes, NVPs, and HTPs, and differences in risk levels [105–107].

Policymakers and researchers can use SAVM to assess the relationship between potential reduced-harm product use and cigarette smoking and evaluate its impact on public health. Following up on Germany’s tobacco control progress, regular monitoring is essential, particularly now as Germany will undergo important changes in tobacco regulation. Germany is one considered a high burden country from tobacco use [108]. It is one of ten countries with the largest number of smokers in Europe and the prevalence of smokers and NVP users has remained relatively stable. Based on the Tobacco Control Scale, in 2019 Germany ranked last in Europe in tobacco control [109]. Germany ratified the World Health Organization Framework Convention on Tobacco Control (WHO-FCTC) [110] but they have yet to implement most of the recommended cigarette-oriented policies [111].

However, in 2021, Germany released the Strategy for Tobacco-Free Germany 2040 (Strategie für ein tabakfreies Deutschland 2040), which proposed the progressive implementation of 10 measures to reduce the use of tobacco, NVPs and other related products to ≤5% of adults and ≤ 2% of adolescents by 2040 [108]. The Germany-SAVM could be a valuable tool to the country in assessing and monitoring policy progress towards proposed intermediate and final targets. As new information is obtained, the model can be modified to guide regulations.

In conclusion, simulation models play an increasingly important role in tobacco control policy decisions, so models must be readily available, easily adaptable, and transparent. SAVM is a generic heuristic simulation tool that is easily adaptable to different countries, has minimal data requirements, and can be used by non-expert modelers. Its framework allows for updates and data modifications to keep up with the scientific advancements in the study of tobacco and NVP use. SAVM-produced estimates can help researchers, policymakers, and other public health stakeholders to analyze the potential population health effects of NVP use. Its specific characteristics allow the user to understand the model better, and synthesize and translate data into a modeling framework that can support future interventions and the the outcomes into more effective interventions to achieve tobacco control goals.

### Supplementary Information

**Additional file 1:** **Supplementary Table 1.** Smoking and Vaping Model, recommended definitions for cigarette use and NVPs use status. **Supplementary Table 2.** SAVM cigarette to nicotine and vaping product use switching rates from the US Population Assessment of Tobacco and Health (PATH) survey 2013-2017. **Supplementary Table 3.** Smoking prevalence (%), validation of Germany-SAVM against the Eurobarometer-Germany, by age and gender, 2014–2020. **Supplementary Figure 1a.** Validation of Germany-SAVM male smoking prevalence vs. German Microcensus 2017. **Supplementary Figure 1b.** Validation of  Germany-SAVM female smoking prevalence  vs. German Microcensus 2017. **Supplementary Figure 2a.** Validation of Germany-SAVM NVP use prevalence vs. the German Study on Tobacco Use (DEBRA). Results for adults ages 18 to 24 years. **Supplementary Figure 2b.** Validation of Germany-SAVM NVP use prevalence vs. the German Study on Tobacco Use (DEBRA). Results for adults ages 25 and above. **Supplementary Table 4.** The Germany SAVM model estimates for all cohorts (ages 18-99) with new births for 2012-2060. NVP risks at 15% those of excess smoking risks. **Supplementary Table 5.** The Smoking and Vaping Model modeling assumption and its implications.

## Data Availability

Data used to parameterize the model are publicly available https://www.gbe-bund.de/gbe/!pkg_olap_tables.prc_set_orientation?p_uid=gastd&p_aid=49431685&p_sprache=E&p_help=2&p_indnr=746&p_ansnr=95402022&p_version=2&D.000=1&D.001=2&D.002=2&D.003=1&D.355=1&D.371=3. The SAVM package and User Guide are made available to the public by the University of Michigan and Georgetown University-Tobacco Center of Regulatory Science (TCORS)-Center for the Assessment of Tobacco Regulation (CAsToR) group upon request at: https://tcors.umich.edu/Resources_Download.php?FileType=SAV_Model
