# Supplementary material for: The smoking and vaping model, A user-friendly model for examining the country-specific impact of nicotine VAPING product use: application to Germany
Source: BMC Public Health. 2023 Nov 21;23:2299. doi: 10.1186/s12889-023-17152-y (PMC10662637; doi:10.1186/s12889-023-17152-y)
Supplement: Supplementary file 1 — Additional file 1: Supplementary Table 1. Smoking and Vaping Model, recommended definitions for cigarette use and NVPs use status. Supplementary Table 2. SAVM cigarette to nicotine and vaping product use switching rates from the US Population Assessment of Tobacco and Health (PATH) survey 2013-2017. Supplementary Table 3. Smoking prevalence (%), validation of Germany-SAVM against the Eurobarometer-Germany, by age and gender, 2014–2020. Supplementary Figure 1a. Validation of Germany-SAVM male smoking prevalence vs. German Microcensus 2017. Supplementary Figure 1b. Validation of Germany-SAVM female smoking prevalence vs. German Microcensus 2017. Supplementary Figure 2a. Validation of Germany-SAVM NVP use prevalence vs. the German Study on Tobacco Use (DEBRA). Results for adults ages 18 to 24 years. Supplementary Figure 2b. Validation of Germany-SAVM NVP use prevalence vs. the German Study on Tobacco Use (DEBRA). Results for adults ages 25 and above. Supplementary Table 4. The Germany SAVM model estimates for all cohorts (ages 18-99) with new births for 2012-2060. NVP risks at 15% those of excess smoking risks. Supplementary Table 5. The Smoking and Vaping Model modeling assumption and its implications. [file 12889_2023_17152_MOESM1_ESM.docx]

**THE SMOKING AND VAPING** **MODEL,**

**A USER-FRIENDLY MODEL FOR EXAMINING THE COUNTRY-SPECIFIC IMPACT OF NICOTINE VAPING PRODUCT USE: APPLICATION TO GERMANY**

**SUPPLEMENTARY INFORMATION**

Luz María Sánchez-Romero^1^, Alex C. Liber^1^, Yameng Li^1^, Zhe Yuan^1^, Jamie Tam^2^, Nargiz Travis^1^, Jihyoun Jeon^3^, Mona Issabakhsh^1^, Rafael Meza^3^, David T. Levy^1^

1. Lombardi Comprehensive Cancer Center, Georgetown University, Washington, DC. USA

2. School of Public Health, Yale University, New Haven, CT. USA

3. Department of Epidemiology, University of Michigan, Ann Arbor, MI. USA

**Corresponding Author**

Luz M. Sánchez-Romero

[ls1364@georgetown.edu](mailto:ls1364@georgetown.edu)

**Supplementary Table 1. Smoking and Vaping Model, recommended definitions for cigarette use and NVPs use status.**

| **Tobacco Use** | **No-NVP Scenario** | **NVP Scenario** |
| --- | --- | --- |
| **Tobacco use states** | | |
| Never users | Smoked less than 100-lifetime cigarettes (herein, never users) | Never use of cigarettes or smoked less than 100-lifetime cigarettes OR never use NVP products or report using NVPs less than a year in duration and less than five days a month. |
| Current Smokers | Smoked 100+ lifetime cigarettes and currently smoke every day or somedays | Smoked 100+ lifetime cigarettes and currently smoke every day or someday  Never users who initiate NVP use even if occasionally and transitions into regular smoking  Current smokers include exclusive cigarettes users and dual-use for cigarettes and NVPs. |
| Exclusive NVP users | -- | Never smoked but currently use NVP regularly (suggested 10+ days in the past 30 days) |
| Former smokers | Smoked 100+ lifetime cigarettes but does not currently smoke | Smoked 100+ lifetime cigarettes but does not currently smoke and/or vape less than 10 days in the past 30 days |
| Former smokers using NVPs | -- | Smokers at ages 35 and above who quit smoking and switch to exclusive NVP use. |
| Former NVP users | -- | An exclusive NVP user who quits NVP use or vaped less than 10 days in the past 30 days. |
| **Transitions between tobacco use states** | | |
| Smoking initiation | A never user who initiates cigarette smoking and becomes a current smoker. | A never user who initiates cigarette smoking and becomes a current smoker with or without any occasional use of NVP.  Includes those who become regular dual users (smoke and regularly use NVPs). |
| NVP initiation | -- | Never user who initiates NVPs in the absence of smoking and becomes an exclusive NVP user AND never users trying smoking, but transitions to regular NVP use. |
| Current smokers to Former smokers using NVPs | -- | A current smoker at 35 years and older who uses NVPs (as a dual user or exclusive user) and soon transitions to NVP use |
| Current smokers to Exclusive NVP user | -- | Current smokers younger than 35 who quit smoking and switch to regular vaping |
| Smoking cessation | Smokers who permanently quit cigarette use for 2 years or more. | Cessation from cigarette use AND dual users who quit both cigarette and NVP use AND smokers who quit smoking and temporarily use NVP |
| NVP cessation | -- | NVP users who successfully quit regular NVP use |

**Supplementary Table 2. SAVM cigarette to nicotine and vaping product use switching rates from the US Population Assessment of Tobacco and Health (PATH) survey 2013-2017**

| Age Group (years) | Females | Males |
| --- | --- | --- |
| 0 to 24 | 2.5% | 4.0% |
| 25 to 34 | 2.0% | 2.5% |
| 35 to 44 | 1.6% | 2.5% |
| 45 to 54 | 1.4% | 1.3% |
| 55 to 64 | 1.4% | 1.2% |
| 65 and older | 1.0% | 0.6% |

**Supplementary Table 3 Smoking prevalence (%), validation of Germany-SAVM against the Eurobarometer-Germany, by age and gender, 2014–2020**

| **Age groups (years)** | **Source** | **2014** | **2017** | **2018** | **2020** | **Relative difference 2014-2018** | **Relative difference 2014-2020** |
| --- | --- | --- | --- | --- | --- | --- | --- |
| ***MALE*** |  |  |  |  |  |  |  |
| 15+ | SAVM | 29.1 | 26.7 | 26.0 | 24.7 | -10.7% | -15.1% |
|  | Eurobarometer | 33.83 | 28.69 | 25.43 | 27.57 | -24.8% | -18.5% |
| 15 - 24 | SAVM | 23.7 | 19.4 | 18.1 | 17.7 | -23.6% | -25.3% |
|  | Eurobarometer | 25.11 | 38.72 | 19.91 | 27.97 | -20.7% | 11.4% |
| 25 - 34 | SAVM | 37.3 | 35.6 | 35.1 | 32.7 | -5.9% | -12.3% |
|  | Eurobarometer | 35.80 | 34.93 | 40.73 | 38.64 | 13.8% | 7.9% |
| 35 - 44 | SAVM | 36.1 | 33.4 | 32.5 | 31 | -10.0% | -14.1% |
|  | Eurobarometer | 46.10 | 34.11 | 22.92 | 36.11 | -50.3% | -21.7% |
| 45 - 54 | SAVM | 33.1 | 30.9 | 30.1 | 28.6 | -9.1% | -13.6% |
|  | Eurobarometer | 43.62 | 28.79 | 31.54 | 33.45 | -27.7% | -23.3% |
| 55 - 64 | SAVM | 28.62 | 27.32 | 26.85 | 25.95 | -6.2% | -9.3% |
|  | Eurobarometer | 36.04 | 30.32 | 27.23 | 25.80 | -24.4% | -28.4% |
| 65+ | SAVM | 18.47 | 16.71 | 16.26 | 15.54 | -12.0% | -15.9% |
|  | Eurobarometer | 17.91 | 13.05 | 13.16 | 10.40 | -26.5% | -41.9% |
| ***FEMALE*** |  |  |  |  |  |  |  |
| 15+ | SAVM | 22.5 | 20.9 | 20.5 | 19.6 | -8.9% | -12.9% |
|  | Eurobarometer | 20.81 | 23.14 | 20.50 | 18.76 | -1.5% | -9.9% |
| 15 - 24 | SAVM | 18.3 | 14.2 | 12.8 | 11.8 | -30.1% | -35.5% |
|  | Eurobarometer | 23.99 | 24.86 | 22.24 | 12.49 | -7.3% | -47.9% |
| 25 - 34 | SAVM | 29.80 | 29.00 | 29.00 | 27.80 | -2.7% | -6.7% |
|  | Eurobarometer | 24.55 | 29.24 | 30.45 | 24.11 | 24.0% | -1.8% |
| 35 - 44 | SAVM | 29.6 | 27.6 | 27 | 25.8 | -8.8% | -12.8% |
|  | Eurobarometer | 24.34 | 29.36 | 30.45 | 28.45 | 25.1% | 16.9% |
| 45 - 54 | SAVM | 27.50 | 25.90 | 25.40 | 24.50 | -7.6% | -10.9% |
|  | Eurobarometer | 28.98 | 33.38 | 18.93 | 20.01 | -34.7% | -31.0% |
| 55 - 64 | SAVM | 23.68 | 22.78 | 22.43 | 21.73 | -5.3% | -8.2% |
|  | Eurobarometer | 19.60 | 17.39 | 20.14 | 22.24 | 2.8% | 13.5% |
| 65+ | SAVM | 13.09 | 12.12 | 11.91 | 11.59 | -9.0% | -11.5% |
|  | Eurobarometer | 10.90 | 12.12 | 10.20 | 10.95 | -6.4% | 0.5% |

Current smokers for Eurobarometer are defined as individuals who answered "currently smoke" cigarettes, cigars, cigarillos, or a pipe

**Supplementary Figure 1a. Validation of Germany-SAVM male smoking prevalence vs. German Microcensus 2017**

Total Population Prevalence

Total PopulationPrevalence

**Supplementary Figure 1b. Validation of Germany-SAVM female smoking prevalence vs. German Microcensus 2017**

Total Population

Total Population

**Supplementary Figure 2a. Validation of Germany-SAVM NVP use prevalence vs. the German Study on Tobacco Use (DEBRA). Results for adults ages 18 to 24 years**

**Supplementary Figure 2b. Validation of Germany-SAVM NVP use prevalence vs. the German Study on Tobacco Use (DEBRA). Results for adults ages 25 and above**

**Supplementary Table 4. The Germany SAVM model estimates for all cohorts (ages 18-99) with new births for 2012-2060. NVP risks at 15% those of excess smoking risks**

| **Year** | | **2012** | **2017** | **2040** | **2060** | **Cumulative*** |
| --- | --- | --- | --- | --- | --- | --- |
| **Male** | | | | | | |
| **No-NVP scenario**** | Smokers | 32.1% | 29.1% | 22.5% | 20.6% | - |
|  | SADs | 111,909 | 108,729 | 99,458 | 86,383 | 4,956,100 |
|  | LYLs | 1,171,679 | 1,189,823 | 890,229 | 671,585 | 46,425,200 |
| **NVP Scenario***** | Smokers | 32.1% | 27.4% | 18.1% | 16.4% | - |
|  | NVP users | 0.0% | 1.3% | 4.4% | 5.5% | - |
|  | FS-NVP users | 0.0% | 0.8% | 1.3% | 0.5% | - |
|  | SVADs | 111,909 | 107,492 | 95,296 | 80,911 | 4,783,026 |
|  | LYLs | 1,171,679 | 1,167,161 | 815,197 | 582,490 | 43,337,384 |
| **Net impact^#^** | Deaths averted | 0 | 1,237 | 4,161 | 5,472 | 173,074 |
|  | LYLs averted | 0 | 22,661 | 75,033 | 89,095 | 3,087,816 |
| **Female** | | | | | | |
| **No-NVP scenario**** | Smokers | 24.7% | 22.6% | 17.7% | 16.1% | - |
|  | SADs | 52,058 | 45,517 | 44,666 | 40,169 | 2,195,568 |
|  | LYLs | 440,936 | 441,049 | 369,645 | 267,812 | 18,462,657 |
| **NVP Scenario***** | Smokers | 24.7% | 21.5% | 15.0% | 13.4% | - |
|  | NVP users | 0.0% | 0.7% | 2.8% | 3.9% | - |
|  | FS-NVP users | 0.0% | 0.6% | 0.8% | 0.3% | - |
|  | SVADs | 52,058 | 44,711 | 42,646 | 38,212 | 2,113,973 |
|  | LYLs | 440,936 | 430,842 | 343,896 | 241,932 | 17,393,894 |
| **Net impact^#^** | Deaths averted | 0 | 806 | 2,020 | 1,957 | 81,594 |
|  | LYLs averted | 0 | 10,206 | 25,749 | 25,880 | 1,068,763 |
| **Both genders** | | | | | | |
| **Net impact^#^** | Deaths averted | 0 | 2,043 | 6,181 | 7,429 | 254,668 |
|  | LYLs averted | 0 | 32,867 | 100,782 | 114,975 | 4,156,579 |
|  | Deaths averted | 0.0% | 1.3% | 4.3% | 5.9% | 3.6% |
|  | LYLs averted | 0.0% | 2.0% | 8.0% | 12.2% | 6.4% |

Results for the NVP scenario are estimated applying a 15% NVP mortality risk of that from smoking and with smoking initiation at 88%, NVP initiation at 25%, and cessation for smoking and NVP at 100% from that of the No-NVP scenario.

NVP = nicotine vaping product, FS-NVP = former smokers nicotine vaping users, SADs= smoking-attributable deaths, SVADs = smoking and vaping attributable deaths, LYL = Life years lost

*Cumulative results include the deaths and life-years lost, which are the sum of attributable deaths or life-years lost over the years 2012-2060.

**No-NVP Scenario refers to values in the absence of NVP use.

***NVP scenario refers to values with NVP use.

# Net impact is the difference between the No-NVP Scenario and NVP Scenario in deaths averted (SADs-SVADs) and LYLs

§ The relative net impact (%) in averted deaths and LYLs are calculated for both genders as:

Deaths averted (%) = Deaths averted /SADs _No-NVP_; LYLs averted (%) = LYLs averted /LYLs _No-NVP_

**Supplementary Table 5. The Smoking and Vaping Model modeling assumption and its implications**

|  | **Assumptions** | **Reasons/Implications** |
| --- | --- | --- |
|  | **General (for both scenarios)** | |
| 1 | The analysis is confined to NVP and cigarette use. No other nicotine products, like smokeless tobacco, cigars, etc. | Simplifies the analysis by avoiding multiple pathways for substitution. These paths may hinder or reinforce the substitution of NVPs for cigarettes. |
| 2 | Population over time is not adjusted based on the changing smoking patterns, i.e., we apply the same population data in NVP and No-NVP Scenarios. | Simplifies the analysis. May understate total deaths due to a larger population in the NVP Scenario due to fewer deaths at early ages. |
| 3 | The ratio of the population death rate between the designated area and the US is the same as the ratio of death rate by smoking status between the designated area and US. | Provides an alternative solution for areas where death rates by smoking status are not available. |
| 4 | When the input population mortality rate is available for only some years within the projection period, we assume the population in future years will decrease/increase by the relative change in the last two years. | Provides a solution for users when the population mortality rates by gender are only available in limited years. |
| 5 | When the input population mortality rate is available only in the initial year, we assume the population in future years will grow with the same trend as in the US. | Provides a solution for users when the population mortality rates by gender are only available in one year. May overestimate the rates in a country with high survival improvement or underestimate it in a country with low survival improvement. |
| 6 | When the input population data is available by age group, we assume the population by single age in this age group is the average of the group. | Provides a solution for users when the population is not available by single age. The overestimation and underestimation within an age group will offset with each other. |
| 7 | When the input population data is available only some years during the projection period, we assume the population in future years will follow the same trend as in the last two years. | Provides a solution for users when the population data is only available in limited years. |
| 8 | When the input population data is available only in the initial year, we assume the population in future years will grow by the same trend as in the US. | Provides a solution for users when the population is only available in one year. May underestimate the population in a high fertility country or overestimate it in a low fertility country. |
| 9 | When the population life expectancy is specified by age group, the model will assume equal life expectancy to all ages within an age group. | Provides a solution for users when the life expectancy by single age and gender is not available. The overestimation and underestimation within an age group will offset each other. |
| 10 | The ratio of the population life expectancy of the user-designated country and the US (in 2016) equals the never smoker life expectancy in the future. | Provides a solution for users when the life expectancy of never smokers is not available. |
| 11 | When the input prevalence of current and former smokers is available by age group, we assume the input value equals the prevalence of the middle age in this age group, and use linear interpolation to estimate the prevalence of other ages between two middle-age points. | Provides a solution for users when the prevalence by single age is not available. May miss the unique fluctuation in the prevalence by single age when the age group is wide (>15 years). |
| 12 | The ratio of the smoking rate in the users' country to that in the US equals the ratio of the initiation rates and reciprocal ratio of the cessation rates. | Provides a solution for users when the initiation rate and the cessation rates by gender are not available. |
|  | **No-NVP Scenario** | |
| 13 | Future initiation and cessation rates reflect levels and trends in past rates | Developed based on age-period-cohort analysis |
| 14 | Future trends reflect No-NVP use | Developed through 2012, before NVP become more widely used in the US. |
|  | **NVP Scenario** | |
| 15 | Smokers switch to NVP users at a rate of σ in the initial year | This process assumes that the switching begins in the first projection year and then follows a pattern in future years that reflects the relative change rate over time. The NVP effects on smoking prevalence are unknown, and there are no established switching rates. |
| 16 | The rate of smokers switching to NVP users σ annually changes exponentially by a constant δ. | The NVP effects on smoking switching over time are unknown. Providing a change rate for simulation is a feasible solution for users to explore the development for future years. |
| 17 | Never smokers initiate smoking at a rate of α_S_ relative to the smoking initiation rate in the No-NVP Scenario. | The age- and gender-specific smoking initiation rate is maintained unless the user designates age and gender-specific variations from those rates. This process assumes that the smoking initiation rate changes in the first projection year and then follows a pattern in future years that reflects the relative change rate over time. The extent of initiation will depend on perceived comparative risks to NVPs and other factors. |
